# Impact of obesity on follicular fluid lipid composition and IVF/ICSI outcomes in Korean women: A lipidomic study

**DOI:** 10.1371/journal.pone.0324511

**Published:** 2025-05-23

**Authors:** Yoo Ra Ko, Min Kyoung Kim, Ji Won Kim, E. Jung Han, Se Jeong Kim, Jae Kyun Park, So Yeon Ahn, Minseo Lee, Surim Oh, Sohyun Hwang, Donghyeon Kim, Hyunjung Jade Lim, Woo Sik Lee

**Affiliations:** 1 Department of Obstetrics and Gynecology, Fertility Center of CHA Gangnam Medical Center, CHA University School of Medicine, Seoul, Republic of Korea; 2 Department of Veterinary Medicine, Konkuk University, Seoul, Republic of Korea; 3 Department of Pathology, CHA Bundang Medical Center, CHA University School of Medicine, Seongnam, Republic of Korea; 4 Department of Life Science, CHA University, Seongnam, Republic of Korea; King Saud University / Zagazig University, EGYPT

## Abstract

**Background:**

Obesity negatively affects reproduction and *in vitro* fertilization (IVF) outcomes. However, its effects on lipid metabolism during embryonic development remain unclear. We examined follicular fluid (FF) lipid composition and body mass index (BMI)-related embryological outcomes in Korean women undergoing IVF/intracytoplasmic sperm injection (ICSI).

**Methods:**

This prospective cohort study included 68 Korean women with infertility without metabolic diseases who underwent IVF/ICSI. Patients were categorized according to the 2022 guidelines of the Korean Society for the Study of Obesity as follows: Group A (obese, BMI ≥ 25 kg/m^2^, n = 28) and Group B (non-obese, BMI < 25 kg/m^2^, n = 40). Liquid chromatography-tandem mass spectrometry (LC-MS) was used to analyze lipids in the FF. Principal component analysis (PCA) and correlation analyses were performed. Embryological outcomes according to the BMI were compared using the QUADE nonparametric analysis of covariance adjusted for age and anti-Müllerian hormone.

**Results:**

LC-MS identified 159 of the 230 lipids in the FF samples. Diacylglycerol (DAG), triacylglycerol (TAG), and acylcarnitine (AC) levels were significantly higher in the obese group; whereas monoacylglycerol (MAG) and plasmenyl phosphatidylcholine levels were lower. PCA explained 38.9% of the variance between the groups. Significant inter-group differences were found in the DAG (adjusted *p* < 0.05) and AC 16:1 (adjusted *p* = 0.0139) levels. BMI and TAG, DAG, and AC levels (adjusted *p *< 0.05) were positively correlated. Obese group had fewer fertilized oocytes (5.07 ± 4.16 vs. 6.65 ± 4.61, *p *= 0.043), cleavage-stage embryos (4.86 ± 4.26 vs. 6.63 ± 4.61, *p *= 0.016), and morula-stage embryos (4.00 ± 4.51 vs. 6.05 ± 5.14, *p *= 0.024).

**Conclusions:**

Obesity alters FF lipid composition in women with infertility undergoing IVF/ICSI, potentially affecting early embryonic development. This study improves our understanding of its effects on the ovarian microenvironment and offers insights into targeted IVF interventions.

## Introduction

According to the Health Insurance Review and Assessment Service-National Health Insurance Service of South Korea, the prevalence of obesity among women aged >20 years will increase by 27.8% by 2021, continuing the decade-long rise. The number of women treated for infertility in Korea is expected to increase from 121,038 in 2018–140,458 in 2022. A recent study revealed that female infertility in Korea was associated with obesity, with an odds ratio of 2.06 (95% confidence interval, 1.61–2.64) compared to women with a normal body mass index (BMI) (18.5 kg/m^2^ ≤ BMI < 25 kg/m^2^) [1]. Since South Korea has the lowest total fertility rate (0.78) among the Organization for Economic Co-operation and Development countries, the rising prevalence of obesity among reproductive-aged women, along with increasing infertility rates, poses a significant social challenge.

In 2021, the American Society for Reproductive Medicine outlined the adverse effects of obesity on reproduction, including impaired ovulatory function, decreased responsiveness to ovarian stimulation, diminished oocyte quality, lower fertilization rates, increased miscarriage rates, and increased risks of maternal and fetal complications during pregnancy [2]. These findings align with those of other studies [1,3–6]. However, obesity has not been reported to significantly affect the clinical or embryological outcomes of *in vitro* fertilization (IVF) [7–8].

Despite the numerous studies on infertility and obesity, the underlying mechanisms remain unclear. Yong et al. reported that obesity affects the hypothalamic-pituitary-ovarian axis, oocyte maturation, embryonic development, and all stages of fetal development, leading to decreased female reproduction [9]. Lipotoxicity is the primary mechanism underlying this impairment, which causes mitochondrial damage in oocytes, an increase in reactive oxygen species, and endoplasmic reticulum stress. These conditions ultimately disrupt hormone production, the formation of cumulus-oocyte complexes (COC), fertilization, and embryonic development [10–15]. Furthermore, obesity causes chronic inflammation by increasing proinflammatory cytokine levels and gene activity [16–18].

As the follicular fluid (FF) represents the immediate microenvironment of the COC, its analysis provides insights into the effects of obesity on IVF outcomes. Alterations in the FF components can reflect in the metabolic levels and influence oocyte development, maturation, and embryo quality [19–21]. Lipid metabolism is essential for oocyte meiosis and folliculogenesis. It provides energy through β-oxidation in mitochondria and functions both as a mediator for cell signaling and as a structural component of cellular membranes and steroid synthesis [15,22–24]. Previous studies have revealed that alterations in FF lipid composition are associated with follicular growth and oocyte development, thereby affecting IVF outcomes [25–30]. Some studies have also sought to identify lipid metabolites in the FF as predictive markers of ovarian stimulation and follicular development [31–32].

However, few studies have investigated the composition of BMI-related lipid metabolites in the FF [12,22,26,33–35]. Triacylglycerols (TAG) and free fatty acids (FA) have been the focus of most studies on lipid metabolites [12,22,25,26,34,35]. While one study revealed no significant change in FF metabolic composition according to BMI, many others have demonstrated a positive correlation between maternal BMI and TAG/FA accumulation, which impairs oocyte development [12,22,25,31]. Additionally, animal *in vitro* maturation studies using lipid supplementation indicate that lipid metabolism with TAG and FA deposition influences oocyte maturation and development [36–38]. However, data on its effects on human oocyte maturation are limited.

Therefore, a comprehensive lipidomics profiling of the FF was conducted to identify changes in metabolites and their effects on oocyte development and embryological outcomes in Korean women with infertility and obesity, defined as a BMI ≥ 25 kg/m^2^ by the Korean Society for the Study of Obesity (KSSO) [39].

## Materials and methods

### Patient selection and follicular fluid collection

This was a single-center prospective cohort study. A total of 68 women without metabolic diseases (such as hyperlipidemia, hypertension, and diabetes mellitus) who underwent IVF/intracytoplasmic sperm injection (ICSI)-embryo transfer between February 2023 and September 2023 were included. The duration of infertility was defined as the period from the initial attempt at conception to the confirmation of clinical pregnancy as reported by the patient through self-examination. Women were divided into two BMI groups according to the KSSO definition. Group A (n = 28) was defined as the obese group with a BMI ≥ 25 kg/m^2^, whereas Group B (n = 40) was defined as the non-obese group with a BMI < 25 kg/m^2^. Transvaginal ultrasound-guided aspiration was used to collect FF from the antral follicles >15 mm during oocyte retrieval.

A stereomicroscope and glass Pasteur pipette were used to retrieve the COC. Following oocyte retrieval, the FF was examined macroscopically. Follicular aspirates exhibiting severe viscosity, lack of clarity, or contamination with endometriotic cysts or the flushing medium were discarded. Only the uncontaminated FF samples were included in the analysis. All samples were preserved at 4 °C and transported to the laboratory on melting ice. This study was approved by the Institutional Review Board of CHA Gangnam Medical Center (IRB approval number: 2022-09-002). Written informed consent was obtained from all the patients.

### Controlled ovarian hyperstimulation (COH) protocol

All patients underwent COH with gonadotropin-releasing hormone (GnRH) agonists or antagonists for pituitary suppression. Stimulation was personalized based on age, ovarian reserve test results, and previous ovarian responses to gonadotropins during stimulation cycles. Ovulation was induced using 250–500 μg of recombinant human chorionic gonadotropin (hCG) (Ovidrel®; Serono, Modugno, Italy) and 0.1–0.2 mg of GnRH agonist (Decapeptyl®; Ferring, Sweden) when at least three follicles with a diameter of at least 17 mm or at least two follicles with a diameter of at least 18 mm were observed in both groups. Oocytes were retrieved 34–36 h after hCG and GnRH agonist administration.

### Assessment of embryological outcomes

Embryological outcomes were assessed as follows: Mature oocytes (metaphase II [MII] oocytes) were defined as those with the first polar body and were subsequently used for ICSI. The oocyte maturation rate was defined as the ratio of MII oocytes to the total number of oocytes retrieved. The presence of two pronuclei and a second polar body 16–18 h after insemination confirmed normal fertilization. The normal fertilization rate was calculated by dividing the number of normally fertilized oocytes by the total number of retrieved oocytes. Total cleavage (CL), morula (MO), and blastocyst (BL) embryo formation rates were determined by dividing the number of embryos at each stage by the number of normally fertilized oocytes. Usable embryos were defined as those available for fresh embryo transfer, pre-implantation genetic testing, or vitrification.

### Reagents

The liquid chromatography-mass spectrometry (LC-MS) grade solvents (isopropanol, methanol, and water), ammonium acetate, butylated hydroxytoluene, chloroform, and methyl *tert*-butyl ether were procured from Sigma-Aldrich (St. Louis, MO, USA). The internal standards were procured from Avanti Polar Lipids (Alabaster, AL, USA) and Sigma-Aldrich (St. Louis, MO, USA) (Table 1).

**Table 1:** Internal standards used for quantification.

No.	Compound	Stock conc. (ng/mL)	Final conc. (ng/mL)	Q_1_*m/z*	Q_3_*m/z*	Dwell time	Collision energy	Company	Catalog No.
1	PC 33:1*d*7 (15:0/18:1)	15060	1506	753.6	184.1	1	-30	Avanti	330707
2	LPC 18:1 *d*7	2380	238	529.4	184.1	2	-25	Avanti	330707
3	PE 33:1 *d*7 (15:0/18:1)	530	53	711.6	570.5	1	-20	Avanti	330707
4	LPE 18:1 *d*7	490	49	487.3	346.3	2	-25	Avanti	330707
5	CE 18:1 *d*7	32910	3291	675.7	369.3	2	-18	Avanti	330707
6	SM 36:2 *d*9 (d18:1/18:1)	2960	296	738.6	184.1	5	-32	Avanti	330707
7	MAG 18:1 *d*7	2000	200	381.3	346.3	7	-18	Avanti	330707
8	DAG 33:1 *d*7 (15:0/18:1)	880	88	605.6	570.5	1	-22	Avanti	330707
9	TAG 48:1 *d*7 (15:0/18:1 (*d*7)/15:0)	5280	528	829.8	570.5	4	-30	Avanti	330707
10	Ceramide 30:1 (d18:0/12:0)	1000	100	657.7	271.3	5	-25	Avanti	860678p
11	Acyl-carnitine 2:0 *d3*	1000	100	207.2	85.1	7	-27	Avanti	330707
12	GalCer 30:1 (d18:1/12:0)	10000	1000	644.5	264.3	1	-35	Avanti	860544
13	Cholesterol *d7*	30000	3000	376.4	147.3	49	-25	Avanti	791108

This table presents the details of the internal standards from Avanti SPLASH Lipid MIX. Internal standards, crucial for ensuring the reproducibility of the analyses, were prepared at a final concentration achieved through a 100-fold dilution, as described above.

Abbreviations: conc., concentration; PC, plasmenyl phosphatidylcholine; LPC, lysophosphatidylcholine; PE, phosphatidylethanolamine; LPE, lysophosphatidylethanolamine; CE, cholesteryl ester; SM, sphingomyelin; MAG, monoacylglycerol; DAG, diacylglycerol; TAG, triacylglycerol; GalCer, galactosylceramides.

### Lipid extraction

**Fig 1:**
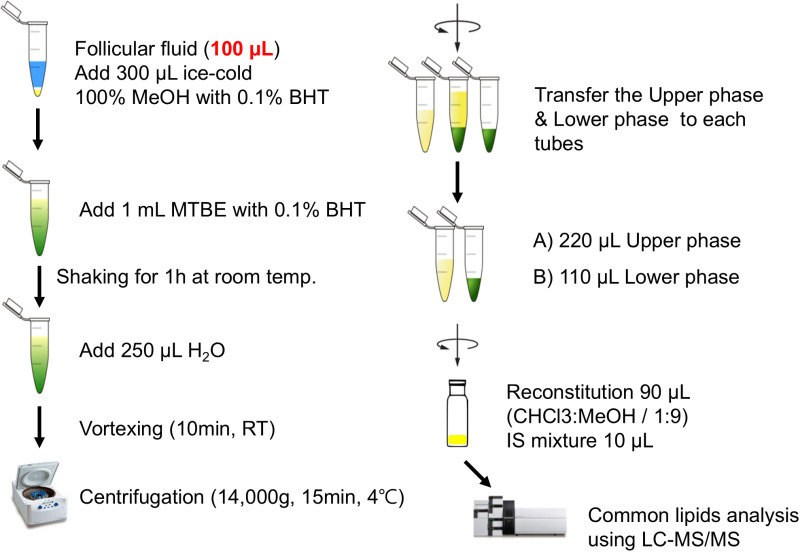
Lipid extraction procedure using the modified Matyash method. This schematic illustrates the process of lipid extraction from the follicular fluid (FF) using the Matyash method with minor modifications. The prepared samples were subjected to lipid analysis using liquified chromatography-mass spectrometry/mass spectrometry (LC-MS/MS).

The Matyash method (with minor modifications) was used for all lipid extractions [40]. In brief, methanol (300 μL) containing 0.1% butylated hydroxytoluene and methyl *tert*-butyl ether (1 mL) was added to the FF (100 μL). After shaking for 1 h at room temperature, 250 μL of water was added, and the mixture vortexed for 10 min. Centrifugation was performed at 14,000 × g (4 °C, 15 min) for phase separation. The upper (220 μL) and lower (110 μL) phases were combined for analysis, and the solvent was evaporated. The sample was then reconstituted in 90 μL of methanol:chloroform (9:1, v/v) solvent with an internal standard mixture (10 μL) (Fig 1). This mixture contained PC 33:1-d7, LPC 18:1-d7, PE 33:1-d7, LPE 18:1-d7, CE 18:1-d7, SM 36:2-d9, MAG 18:1-d7, DAG 33:1-d7, TAG 48:1-d7, cholesterol-d7, ceramide 42:1-d7, AC 2:0-d3, and galactosylceramides 30:1.

### LC-MS analysis

LC-MS/MS (Shimadzu LCMS 8060 system, Shimadzu, Mass Spectrometry Based Convergence Research Institute, Kyungpook National University, Kyoto, Japan) was used to analyze all samples. Kinetex C18 column (100 × 2.1 mm, 2.6 μm particle size, Phenomenex, Torrance, CA, USA) was used to separate them. Mobile phases A and B consisted of 10 mM ammonium acetate in water/methanol (1:9, v/v) and 10 mM ammonium acetate in methanol/isopropanol (1:1, v/v), respectively. The gradient elution conditions were: 30% B (0 min), 95% B (15 min), 95% B (20 min), and 30% B (25 min). The sample injection volume was 1 μL, and the flow rate was maintained at 0.2 mL/min. The mass operating conditions were as follows: desolvation temperature, 250 °C; heat block temperature, 400 °C; spray voltage, 4 kV; drying gas (nitrogen) flow rate, 10 L/min; collision gas, argon; nebulizing gas (nitrogen) flow rate, 3 L/min; collision gas pressure, 230 kPa; and detector voltage, 1.82 kV. Selected reactions were monitored. Three samples were randomly selected from each group for the preliminary experiment, yielding 230 lipid species. These detected lipids were then analyzed. In the final analysis, 159 of 230 lipids (Table 2 and S1-S5 Tables) in the FF samples were quantified after validating the assay by confirming the presence of lipid species with a coefficient of variation <30% of the quality control (QC) sample.

**Table 2:** Selected reaction monitoring table.

Type		Number	
Phospholipid	Lysophosphatidylcholine	14	87
	Lysophosphatidylethanolamine	10	
	Phosphatidylcholine (PC)	24	
	Phosphatidylethanolamine (PE)	5	
	Plasmenyl PC	16	
	Plasmanyl PC	2	
	Plasmecyl PE	16	
Sphingolipid	Sphingomyelin	10	11
	Ceramide	1	
Sterol lipid	Cholesterol	1	10
	Cholesteryl ester	9	
Carnitine	Acylcarnitine	8	8
Neutral lipid	Monoacylglycerol	1	43
	Diacylglycerol	5	
	Triacylglycerol	37	
Total			159

### Statistical analysis

The data for all detected peaks, including the peak areas and retention times, were exported to an Excel file for lipidomic analysis. The QC samples were prepared by mixing equal amounts of lipid extracts from all samples. Lipidomics data were analyzed using the Student’s t-test, Welch’s t-test, or Wilcoxon rank-sum test based on the underlying assumptions of the parametric tests, including normality and equality of variance. The analyses were performed using the stats R package. Principal component analysis (PCA) was performed using the prcomp function in the stats R package. Correlation analyses were performed using the rcorr function in the HMISC R package. The p-values were adjusted for multiple comparisons using the Benjamini-Hochberg method with the p.adjust function from the stats R package. PCA and scatter plots were generated using the ggplot2 package in R.

Inter-group differences in clinical outcomes were assessed using the Student’s t-test, Welch’s t-test, or Wilcoxon rank-sum test for continuous variables, depending on the underlying assumptions of the parametric tests. Categorical variables are presented as percentages, and differences in these variables were analyzed using the Chi-squared test or Fisher’s exact test. Due to unequal sample sizes, analysis of covariance or Quade’s analysis of covariance was used to evaluate the differences between the BMI groups and embryological outcomes of IVF/ICSI, adjusting for potential confounders. Age and anti-Müllerian hormone (AMH) levels were selected as covariates based on clinical knowledge. P-values < 0.05 were considered statistically significant. All analyses were performed using R software version 4.4.1.

## Results

### Participant characteristics

The clinical characteristics of the 68 patients included in this study are presented in Table 3. Group A (obese group, BMI ≥ 25 kg/m^2^) comprised 41.2% (n = 28) of the participants, whereas Group B (non-obese group, BMI < 25 kg/m^2^) comprised 58.8% (n = 40). The mean BMIs for the two groups were significantly different (Group A: 27.78 ± 2.12 kg/m^2^; Group B:22.00 ± 2.37 kg/m^2^; *p *< 0.001). The mean total serum cholesterol levels were significantly different between groups (Group A: 213.78 ± 36.64 mg/dL; Group B: 195.24 ± 0.78 mg/dL; *p *= 0.031). Additionally, the total cholesterol levels were positively correlated with the BMI (*p *= 0.002); a unit-increase in BMI corresponded to an estimated increase of 3.601 mg/dL in the serum cholesterol levels. Other variables, including age, cause of infertility, antral follicle count, basal follicle-stimulating hormone levels, AMH levels, infertility duration, and number of previous IVF attempts, were not significantly different between the groups. Other cycle variables, including stimulation protocol, total gonadotropin dose, duration of stimulation, estradiol level on the triggering day, type of trigger, and fertilization type, did not show significant differences between the groups (Table 4).

**Table 3:** Clinical characteristics of patients categorized according to BMI.

Variables	Group A (BMI ≥ 25 kg/m^2^) (n = 28)	Group B (BMI < 25 kg/m^2^) (n = 40)	*p*
Female age, years	37.82 ± 4.14	38.13 ± 3.33	0.739
Maternal BMI (kg/m^2^)	27.78 ± 2.12	22.00 ± 2.37	< 0.001
Cause of infertility (n)			0.088
Male factor	5 (17.9%)	9 (22.5%)	
Tubal factor	1 (3.6%)	1 (2.5%)	
Ovulation disorder (e.g., PCOS)	5 (17.9%)	1 (2.5%)	
Endometriosis	1 (3.6%)	2 (5.0%)	
Uterine factor	1 (3.6%)	8 (20.0%)	
Diminished ovarian reserve	10 (35.7%)	8 (20.0%)	
Unexplained	5 (17.9%)	11 (27.5%)	
Secondary infertility	1 (3.6%)	6 (15.0%)	0.226
Total cholesterol level (mg/dL)	213.78 ± 36.64	195.24 ± 30.78	0.031
AFC count	13.07 ± 8.21	11.48 ± 6.16	0.689
Basal FSH level (mIU/mL)	7.79 ± 2.65	8.14 ± 3.51	0.980
AMH level (ng/mL)	2.91 ± 3.05	2.09 ± 1.54	0.737
Infertility duration (months)	37.54 ± 24.67	29.33 ± 24.38	0.050
Number of previous IVF attempts (n)	2.14 ± 2.76	1.78 ± 2.14	0.802

BMI, body mass index; PCOS, polycystic ovary syndrome; AFC, antral follicle count; AMH, anti- Müllerian hormone; FSH, follicle-stimulating hormone; IVF, *in vitro* fertilization; n, number of cycles; NS, not significant (*p* > 0.05). Values are presented as mean ± standard deviation

**Table 4:** Cycle characteristics of participants categorized according to BMI.

Variables	Group A (BMI ≥ 25 kg/m^2^) (n = 28)	Group B (BMI < 25 kg/m^2^) (n = 40)	*p*
Stimulation protocol (n)			1.00
GnRH long agonist	3 (10.7%)	5 (12.5%)	
GnRH antagonist	25 (89.3%)	35 (87.5%)	
Total gonadotropin (IU)	2021.04 ± 729.00	2212 ± 817,60	0.326
Duration of simulation (days)	9.14 ± 2.29	9.30 ± 1.92	0.924
Estradiol on triggering day (pg/mL)	1387.00 ± 1146.86	2124.06 ± 1985.37	0.053
Trigger			0.778
Ovi	4 (14.3%)	8 (20.0%)	
Ovi + Deca	22 (78.6%)	28 (70.0%)	
Deca	2 (7.1%)	4 (10.0%)	
Fertilization type (n)			1.00
IVF	4 (14.3%)	5 (12.5%)	
ICSI	24 (85.7%)	35 (87.5%)	

n, number of cycles; BMI, body mass index; GnRH, gonadotropin-releasing hormone; Ovi, oviderel; Deca, decapeptyl; IVF, *in vitro* fertilization; ICSI, intracytoplasmic sperm injection. Values are presented as mean ± standard deviation or as number (%).

### Lipid composition analysis

**Fig 2:**
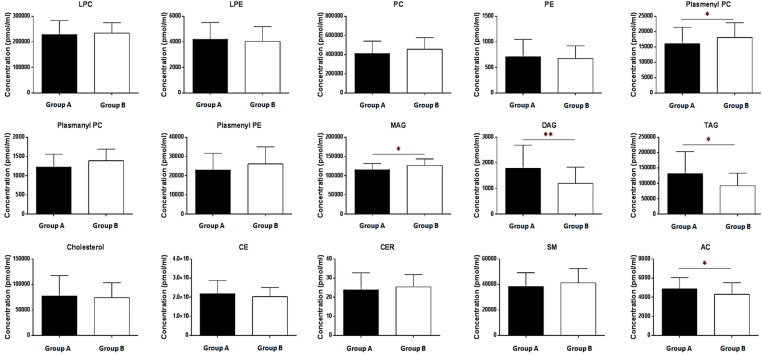
Lipid concentration differences (pmol/mL) between the BMI groups. The bar graphs show the differences in lipid concentrations between the BMI groups. The lipid concentration (pmol/mL) was normalized to the volume. Different letters indicate significant differences compared with the control group [(*) *p* < 0.05, (**) *p* < 0.01], as determined using the t-test. Data are shown as mean ± standard deviation (n = 28 and n = 40). BMI, body mass index; LPC, Lysophosphatidylcholine; LPE, lysophosphatidylethanolamine; PC, phosphatidylcholine; PE, phosphatidylethanolamine; MAG, monoacylglycerol; DAG, diacylglycerol; TAG, triacylglycerol; SM, sphingomyelin; CER, ceramide; CE, cholesteryl ester; AC, acylcarnitine; Group A, obese group; Group B, non-obese group.

**Fig 3:**
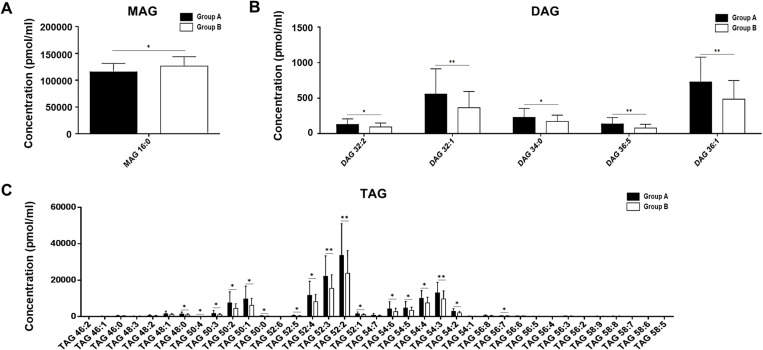
Subgroup analysis of concentrations of neutral lipids (pmol/mL) which showed significant differences between BMI groups. The bar graphs show the lipid subgroup concentration differences between the BMI groups and (A) MAG, (B) DAG, and (C) TAG. The lipid concentration (pmol/mL) was normalized to the volume. Different letters indicate significant differences compared with the control group [(*) *p *< 0.05, (**) *p *< 0.01], as determined by the t-test. The data shown as mean ± standard deviation (n = 28 and n = 40). BMI, body mass index; MAG, monoacylglycerol; DAG, diacylglycerol; TAG, triacylglycerol.

A total of 159 of the 230 lipids (Table 2 and Additional File 1, S1–S5 Tables) in the FF samples were quantified. The assay was validated by confirming that lipid species with coefficient of variation <30% in the QC sample exceeded 70% (83.0%) of the total lipid species [41]. Significant differences were observed between the two groups in all neutral lipid compositions (Figs 2 and 3). Group A exhibited markedly higher concentrations of TAG than Group B (Group A: 132,443.3 ± 71,011.6 pmol/mL; Group B: 92,298.1 ± 41,084.8 pmol/mL; *p *= 0.0102; Fig 2). Similarly, DAG levels were also elevated in Group A relative to Group B (Group A: 1,788.4 ± 903.0 pmol/mL; Group B: 1,198.0 ± 636.9 pmol/mL; *p *= 0.0014; Fig 2). However, MAG levels (16:0) demonstrated a negative correlation with the BMI (Group A: 115,531.2 ± 16,058.7 pmol/mL; Group B = 126,500.0 ± 17,281.2 pmol/mL; *p =* 0.0149; Fig 3A).

Additionally, a notable difference was observed in the composition of neutral lipids between groups. In Group A, the compositions of TAG and DAG were higher at 53.02% and 0.72%, respectively, compared to Group B, which had compositions of 41.19% and 0.54%, respectively. Conversely, Group A exhibited lower MAG content (46.26%) than Group B (57.27%).

Further analysis was conducted on the DAG lipid chains, covering five DAGs (32:2, 32:1, 34:0, 36:5, and 36:1), all showing statistical significance, with mean concentrations positively correlated with the groups (Fig 3B). Additionally, the analysis of 37 TAGs revealed significant differences in TAGs 48:0, 50:4, 50:3, 50:2, 50:1, 50:0, 52:5, 54:4, 52:3, 52:2, 52:1, 54:6, 54:5, 54:4, 54:3, 54:2, and 56:7 (Fig 3C).

**Fig 4:**
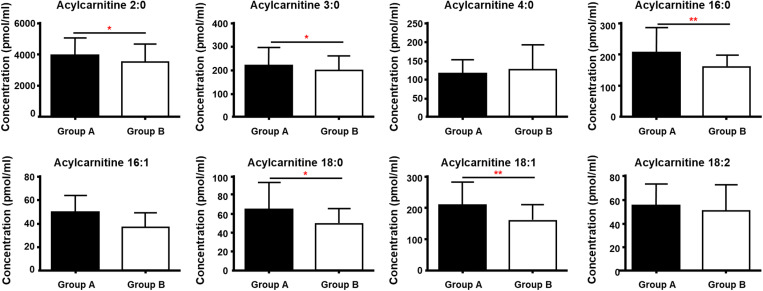
Acylcarnitine subgroup concentration differences (pmol/mL) between BMI groups. These bar graphs show the concentration differences between the BMI groups and the AC subgroup. The lipid concentration (pmol/mL) was normalized to the volume. Different letters indicate significant differences compared with the control group [(*) *p *< 0.05, (**) *p *< 0.01], as determined by the t-test. The data shown as mean ± standard deviation (n = 28 and n = 40). BMI, body mass index; AC, acylcarnitine.

Additionally, total AC levels were higher in group A than in group B (Group A: 4878.1 ± 1179.3 pmol/mL; Group B: 4302.1 ± 1216.0; *p *= 0.0222; Fig 2). Chain analysis revealed significant differences in C2:0, C3:0, C16:0, C18:0, and C18:1 levels, all of which were higher in Group A than in Group B (Fig 4).

Of the phospholipid family, only plasmenyl PC demonstrated a statistically significant inverse relationship with the BMI (Group A: 1,6093.3 ± 5323.7 pmol/mL; Group B: 1,8121.9 ± 4797.2 pmol/mL; *p *= 0.0423; Fig 2). Further analysis of plasmenyl PC chains revealed that plasmenyl 34:0, 36:2, 40:7, and 40:4 showed significantly higher levels in Group B. No significant differences were observed between the groups in other lipids, such as sphingolipids (sphingomyelin and ceramide) or sterol lipids (cholesterol and cholesteryl ester) (Fig 2).

Adjusted *p*-value analysis revealed that, among the total lipids, only DAG showed a significant difference between groups. Among the total lipid chains, only AC 16:0 differed significantly between groups (adjusted p < 0.05).

**Fig 5:**
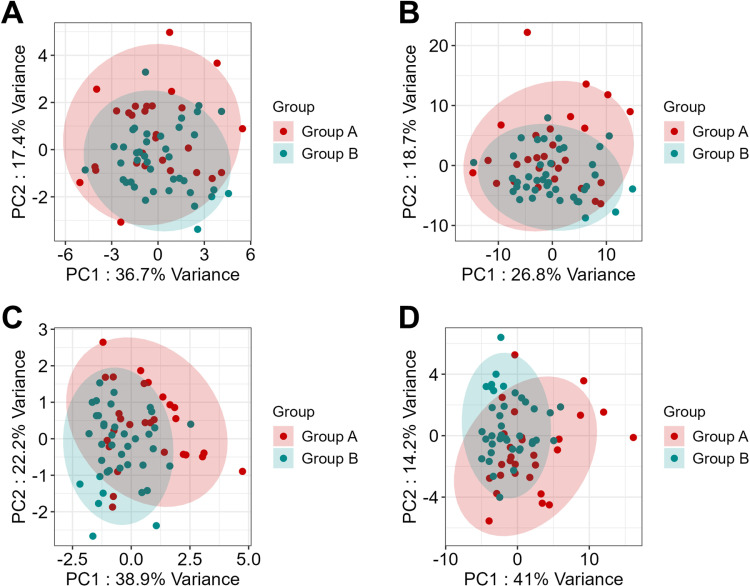
PCA plots for lipid and lipid chain concentrations. The PCA plots show that the distributions of (A) all lipids, (B) all lipid chains, (C) lipids, and (D) lipid chains were significantly different between the two groups. The light green and red areas represent the 95% confidence intervals for each group. PCA, principal component analysis.

The lipid and lipid chain concentration data were not clearly separated between the obese and non-obese groups in the PCA (Figs 5A and 5B). When analyzing only the lipids and lipid chains that showed a significant difference in abundance between the groups (*p *< 0.05), some differences were observed. The variance explained by the first principal component increased significantly from 36.7% to 38.9% and from 26.8% to 41.0% for lipids and lipid chains, respectively, indicating that these discriminatory lipids or lipid chains could help distinguish between the groups (Figs 5C and 5D).

**Fig 6:**
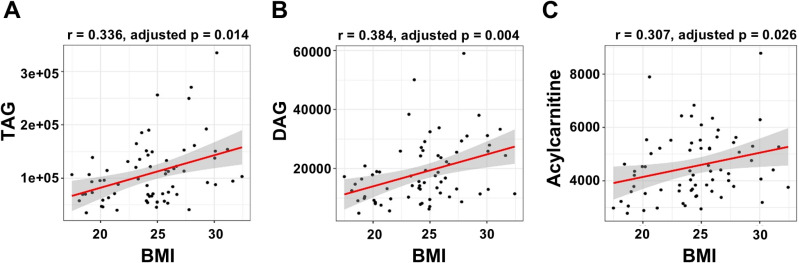
Correlation between lipid concentrations and BMI. These scatter plots show a positive correlation between the BMI and (A) TAG, (B) DAG, and (C) AC levels, respectively. The red line represents the linear regression fit of each data point and the gray area indicates the 95% confidence interval of the regression fit. The correlation coefficient (r) was calculated using Spearman’s correlation method. BMI, body mass index; TAG, triacylglycerol; DAG, diacylglycerol; AC, acylcarnitine.

**Fig 7:**
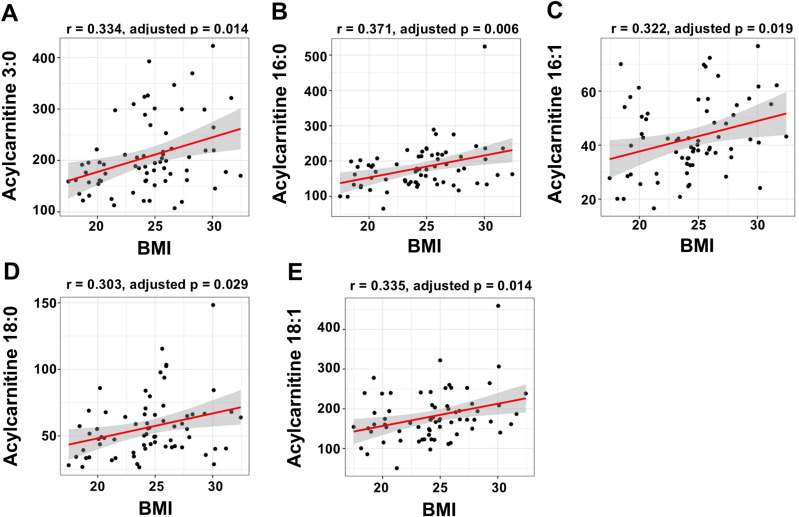
Correlation between acylcarnitine subgroup concentrations and BMI. Scatter plots show a positive correlation between the BMI and (A) AC 3:0, (B) AC 16:0, (C) AC 16:1, (D) AC 18:0, and (E) AC 18:1. The red line represents the linear regression fit of each data point and the gray area indicates the 95% confidence interval of the regression fit. The correlation coefficient (r) was calculated using Spearman’s correlation method. BMI, body mass index; AC, acylcarnitine.

Correlation analysis revealed a significant positive correlation between BMI and TAG, DAG, and AC levels (adjusted *p *< 0.05; Fig 6). Further analysis of AC chains showed that AC 3:0, 16:0, 16:1, 18:0, and 18:1 levels were significantly positively associated with the BMI (adjusted *p *= 0.014, 0.006, 0.019, 0.029, and 0.014, respectively; Fig 7).

### Embryological outcomes

After adjusting for maternal age and AMH, which had the most significant impact on embryological outcomes, notable differences were observed in the numbers of fertilized oocytes, CL-stage embryos, and MO-stage embryos between Groups A and B. The total number of oocytes retrieved during the COH cycle was comparable between groups. However, in comparison to Group B, Group A had significantly fewer fertilized oocytes (Group A: 5.07 ± 4.16; Group B: 6.65 ± 4.61; *p *= 0.043), as well as fewer CL-stage (Group A: 4.86 ± 4.26; Group B: 6.63 ± 4.61; *p *= 0.016) and MO-stage (Group A: 4.00 ± 4.51; Group B: 6.05 ± 5.14; *p *= 0.024) embryos. Nevertheless, Group A exhibited lower values across various embryological outcomes, including the number of mature oocytes retrieved, oocyte maturation rate, fertilization rate, CL-embryo formation rate, MO-embryo formation rate, number of total BL-stage embryos, BL formation rate, number of usable embryos, and usable embryo rate; however, these differences were not statistically significant (Table 5).

**Table 5:** Comparison of IVF/embryological outcomes and clinical outcomes according to BMI groups with adjustment for age and AMH.

Variables	Group A (BMI ≥ 25 kg/m^2^) (n = 28)	Group B (BMI < 25 kg/m^2^) (n = 40)	*p*
Oocytes retrieved in stimulated cycles (n)	10.50 ± 8.86	10.50 ± 6.58	0.241
Mature oocytes retrieved in stimulated cycles (n)	7.82 ± 6.17	8.63 ± 5.61	0.197
Oocyte maturation rate	75.9%	80.9%	0.517
Fertilized (2PN) oocyte (n)	5.07 ± 4.16	6.65 ± 4.61	0.043
Fertilization rate	48.7%	61.6%	0.098
Total cleavage-stage embryo (n)	4.86 ± 4.26	6.63 ± 4.61	0.016
Cleaved embryo formation rate	95.3%	99.6%	0.276
Total morula-stage embryo (n)	4.00 ± 4.51	6.05 ± 5.14	0.024
Morula-stage embryo formation rate	78.0%	91.6%	0.076
Total blastocyst-stage embryo (n)	1.64 ± 1.91	2.38 ± 2.32	0.060
Blastocyst formation rate	32.3%	38.9%	0.180
Usable embryo (n)	1.30 ± 1.33	1.85 ± 1.56	0.075
Usable embryo rate	25.2%	27.2%	0.817

Data are presented either as mean ± standard deviation or as percentages.

Abbreviations: IVF, *in vitro* fertilization; BMI, body mass index; AMH, anti-Müllerian hormone.

## Discussion

Lipid metabolic pathways, including the TAG breakdown, FA oxidation, and cholesterol uptake pathways, are active within the COC. The lipid and FA compositions of the FF vary with the developmental stage of the ovarian follicle, reflecting differences in lipid function [15,27]. Because oocytes have low glycolytic activity, they primarily depend on internal lipid storage within the COC and external sources such as FAs and amino acids from the FF for energy [15,22,26]. This metabolically quiescent state continues until the early CL stage due to limited glycolytic activity [42]. Recent proteomics and transcriptomics studies have highlighted the essential role of lipid metabolism in folliculogenesis and oocyte maturation [42–44]. Lipid metabolism influences chromatin dynamics and gene expression, affecting both nuclear and cytoplasmic maturation of oocytes and early embryonic development [43]. As COH promotes the development of additional follicles and maturation of more oocytes, lipid metabolism becomes increasingly crucial.

Here, we report that neutral lipids, which are the primary sources of lipid metabolism, exhibited the most significant differences between the obese and non-obese groups. Consistent with other studies, the TAG levels were markedly higher in the obese group than in the non-obese group, indicating a significant positive correlation with the BMI [12,22,25,26,34,35]. Additionally, we observed significant differences in the numbers of fertilized oocytes, CL-stage embryos, and MO-stage embryos between the groups. This aligns with existing studies indicating that elevated triglyceride (TG) levels in the FF are associated with oocyte CL failure and reduced embryo viability [12,22,31,45].

In addition to elevated TAG levels, our analysis revealed that the obese group exhibited higher DAG levels, suggesting a statistically significant positive correlation with the BMI. In contrast, the obese group exhibited lower MAG levels than the non-obese group.

Lipolysis in the COC involves the action of lipases, which are likely to be regulated during maturation. Lipolysis begins with the action of adipose TG lipase, which hydrolyzes TAG to DAG and FA. Subsequently, a hormone-sensitive lipase breaks down DAG into MAG, which is ultimately converted into FA and glycerol by MAG lipase [46]. Our results suggest that the initial steps of lipolysis in the FF are less effective in the obese group, as indicated by the accumulation of DAG, an intermediate metabolite, and reduced levels of MAG, the final metabolite of lipolysis. Although the mechanisms linking obesity and ineffective lipolysis within the COC are not well-established, a study on adipocytes showed decreased catecholamine-stimulated lipolysis and an antilipolytic effect of insulin in obesity [47].

Furthermore, incomplete lipolysis leads to the accumulation of DAG, a critical signaling molecule, during fertilization and early embryonic development. After sperm and oocyte fusion, DAG is generated by sperm-specific phospholipase C zeta hydrolyzing phosphatidylinositol 4,5-bisphosphate [48]. DAG then activates protein kinase C (PKC), which regulates meiosis resumption, cytoskeletal dynamics, and the mitogen-activated protein kinase pathway, which are essential for oocyte maturation, fertilization, and early embryonic development [49–51].

However, research on mouse COC revealed that high DAG concentrations eventually reduce fertilization, as excessive DAG in phospholipase C zeta-injected eggs can trigger PKC-mediated Ca^2+^ influx, leading to Ca^2+^ overload and disrupted homeostasis [51]. Microinjection of DAG into oocytes also results in developmental arrest at the 4- to 68-cell stage [52]. Additionally, prolonged DAG activation in T cells downregulates PKC isoforms [53–55]. Studies in human liver and muscle tissues have indicated that elevated DAG levels activate PKC, disrupt insulin signaling, and contribute to insulin resistance and lipotoxicity [56–58]. Although no study has specifically examined the effects of DAG on human oocytes, our findings suggest that elevated DAG levels may contribute to poor fertilization and embryonic outcomes.

AC acts as the transportation site for FAs across the mitochondrial membrane and undergoes β-oxidation, which is a significant energy-producing process [55]. Given that oocytes and early embryos exhibit low glycolytic activity, β-oxidation is crucial during these stages. The accumulation of ACs in the obesity group indicates incomplete β-oxidation and, by extension, an inefficient FA oxidation process. This inefficiency can lead to decreased adenosine triphosphate levels, which may disrupt meiotic spindle formation and lead to failure of chromosomal segregation, maturation, and fertilization [59]. These disruptions are characterized by a lower number of fertilized oocytes and poor early embryological outcomes. Furthermore, long-chain ACs are correlated with gonadotropin-induced T levels, resulting in hormonal imbalances [24]. Additionally, a metabolomics study revealed that elevated plasma levels of medium- and long-chain ACs are associated with an increased risk of diabetic cardiomyopathy, which exacerbates myocardial lipotoxicity [60]. Thus, our detailed analysis of individual AC chains provides valuable insights, suggesting that the accumulation of ACs may be detrimental to embryological outcomes.

In this study, we utilized a large-scale untargeted lipidomic approach to comprehensively identify lipidomic profiles and analyze a diverse range of lipid classes and subtypes. A few human studies have explored the relationship between BMI, FF lipidomes, and embryological outcomes. Although the dynamics of lipid metabolism during oocyte maturation, fertilization, and early embryonic development in humans remain unclear, our findings highlight significant differences in the composition of follicular fluid lipids and their influence on embryological outcomes. However, this study has several limitations.

While we excluded patients with underlying metabolic diseases, we included three individuals with endometriosis and six with polycystic ovary syndrome, which may influence the local FF microenvironment. However, the number of patients with these conditions was small. Additionally, only metabolically healthy patients with polycystic ovary syndrome and those with mild endometriosis (lesions ≤ 1.5 cm) were included in our analysis. Few studies have suggested a relationship between FF lipid changes, age, and ovarian reserve [61,62]. However, the lack of significant differences in the mean AMH levels or age between the obese and non-obese groups suggests that age possibly had minimal impact on our findings. Since we focused on patients without hyperlipidemia, we only measured total serum cholesterol levels. Given that the composition of FF metabolites originates from the blood and COC, additional lipidomic analyses, including LDL, HDL, and TGs, would further enhance our understanding of how obesity affects the FF lipidome. The small sample size is another limitation, and it is challenging to determine whether the observed results are a cause or an effect. Consequently, studies with larger sample sizes are warranted to validate our findings, and further mechanistic studies are required to elucidate the underlying pathways. In addition, multicenter studies involving diverse populations are essential to confirm the generalizability of our findings across different clinical settings.

## Conclusions

This is the first large-scale lipidomic study involving the FF to identify differences in metabolic profiles in the ovarian microenvironment associated with obesity in Korean women with infertility. We observed significant differences in the number of fertilized oocytes, CL-stage embryos, and MO-stage embryos, indicating altered embryonic development in the obese group. Analysis of the FF lipid composition provides valuable insights into the metabolic state and microenvironment of the COC. The accumulation of TAGs and DAGs, coupled with lower levels of MAGs and higher levels of ACs in the obese group, indicated inefficient lipid metabolism with insufficient energy production and potential lipotoxicity. This lipid imbalance may compromise early embryological outcomes in patients with obesity. Our results provide crucial insights into the importance of balanced lipid metabolism under conditions of increased energy demand during early embryonic development. This study provides a deeper understanding of the pathophysiology of lipid metabolism and obesity during early embryonic development. Mechanistic studies with larger sample sizes are needed to understand obesity-associated lipid metabolism and develop targeted interventions to improve fertility outcomes in women with obesity. Moreover, multicenter studies are necessary to enhance the external validity of our results and ensure their applicability to broader patient populations.

## Supporting information

S1 Table SRM condition of phospholipids in lipid droplet by LC-MS/MS. SRM, selected reaction monitoring; LC, liquid chromatography; MS, mass spectrometry; LPC, lysophosphatidylcholine; LPE, lysophosphatidylethanolamine; PC, phosphatidylcholine; PE, phosphatidylethanolamine
(DOCX)

S2 Table SRM condition of neutral lipids in lipid droplet by LC-MS/MS. SRM, selected reaction monitoring; LC, liquid chromatography; MS, mass spectrometry; MAG, monoacylglycerol; DAG, diacylglycerol; TAG, triacylglycerol.
(DOCX)

S3 Table SRM condition of sphingolipid in lipid droplet by LC-MS/MS. SRM, selected reaction monitoring; LC, liquid chromatography; MS, mass spectrometry; SM, sphingomyelin; CER, ceramide.
(DOCX)

S4 Table SRM condition of sterol lipid in lipid droplet by LC-MS/MS. SRM, selected reaction monitoring; LC, liquid chromatography; MS, mass spectrometry; CE, cholesteryl ester.
(DOCX)

S5 Table SRM condition of carnitine in lipid droplet by LC-MS/MS. SRM, selected reaction monitoring; LC, liquid chromatography; MS, mass spectrometry.
(DOCX)

## Abbreviations

IVF: *In* vitro fertilization
ICSI: Intracytoplasmic sperm injection
BMI: Body mass index
COH: Controlled ovarian hyperstimulation
COC: Cumulus–oocyte complexes
FF: Follicular fluid
KSSO: Korean Society for the Study of Obesity
LC-MS: Liquid chromatography–tandem mass spectrometry
hCG: Human chorionic gonadotropin
QC: Quality control
PKC: Protein kinase C
PCA: Principal component analysis
MAG: Monoacylglycerol
DAG: Diacylglycerol
TAG: Triacylglycerol
AC: Acylcarnitine
PC: Phosphatidylcholine
TG: Triglyceride
FA: Fatty acid
CL: Cleavage
MO: Morula
BL: Blastocyst.

## References

[1] Lee J, Choo C-W, Moon KY, Lyu SW, Kim H, Lee JY, et al. Risk factors for infertility in Korean Women. J Korean Med Sci. 2024;39(10):e85. doi: 10.3346/jkms.2024.39.e85 38501182 PMC10948255

[2] Obesity and reproduction: a committee opinion. Fertil Steril. 2021;116(5):1266–85. 34583840 10.1016/j.fertnstert.2021.08.018

[3] García-Ferreyra J, Carpio J, Zambrano M, Valdivieso-Mejía P, Valdivieso-Rivera P. Overweight and obesity significantly reduce pregnancy, implantation, and live birth rates in women undergoing in vitro fertilization procedures. JBRA Assist Reprod. 2021;25(3):394–402. 33710838 10.5935/1518-0557.20200105 PMC8312282

[4] Wuytack F, Devlieger R, Ameye L, Corcoran P, Fitzgerald AP, Ombelet W. Impact of female obesity and assisted reproduction on uncomplicated pregnancies and healthy births: a study of 428 336 births in Flanders. Hum Reprod. 2023;38(1):156–67. 36256863 10.1093/humrep/deac221

[5] Rafael F, Rodrigues M, Bellver J, Canelas-Pais M, Garrido N, Garcia-Velasco J. The combined effect of BMI and age on ART outcomes. Hum Reprod. 2023;38(5):886–94. 36928306 10.1093/humrep/dead042

[6] Tang J, Xu Y, Wang Z, Ji X, Qiu Q, Mai Z. Association between metabolic healthy obesity and female infertility: the national health and nutrition examination survey, 2013-2020. BMC Public Health. 2023;23(1):1524. 37563562 10.1186/s12889-023-16397-x PMC10416469

[7] Bellver J, Brandão P, Alegre L, Meseguer M. Blastocyst formation is similar in obese and normal weight women: a morphokinetic study. Hum Reprod. 2021;36(12):3062–73. 34601596 10.1093/humrep/deab212

[8] Kidera N, Ishikawa T, Kawamura T, Miyasaka N. Maternal body mass index is not associated with assisted reproductive technology outcomes. Sci Rep. 2023;13(1):14817. doi: 10.1038/s41598-023-41780-4 37684397 PMC10491661

[9] Yong W, Wang J, Leng Y, Li L, Wang H. Role of obesity in female reproduction. Int J Med Sci. 2023;20(3):366–75. doi: 10.7150/ijms.80189 36860674 PMC9969507

[10] Sørensen TIA, Virtue S, Vidal-Puig A. Obesity as a clinical and public health problem: is there a need for a new definition based on lipotoxicity effects?. Biochim Biophys Acta. 2010;1801(3):400–4. doi: 10.1016/j.bbalip.2009.12.011 20045743

[11] Wu LL-Y, Dunning KR, Yang X, Russell DL, Lane M, Norman RJ, et al. High-fat diet causes lipotoxicity responses in cumulus-oocyte complexes and decreased fertilization rates. Endocrinology. 2010;151(11):5438–45. doi: 10.1210/en.2010-0551 20861227

[12] Si C, Wang N, Wang M, Liu Y, Niu Z, Ding Z. TMT-based proteomic and bioinformatic analyses of human granulosa cells from obese and normal-weight female subjects. Reprod Biol Endocrinol. 2021;19(1):75. doi: 10.1186/s12958-021-00760-x 34016141 PMC8135161

[13] Dick KJ, Nelson CP, Tsaprouni L, Sandling JK, Aïssi D, Wahl S, et al. DNA methylation and body-mass index: a genome-wide analysis. Lancet. 2014;383(9933):1990–8. doi: 10.1016/S0140-6736(13)62674-4 24630777

[14] Crujeiras AB, Casanueva FF. Obesity and the reproductive system disorders: epigenetics as a potential bridge. Hum Reprod Update. 2015;21(2):249–61. doi: 10.1093/humupd/dmu060 25413685

[15] Liu T, Qu J, Tian M, Yang R, Song X, Li R, et al. Lipid metabolic process involved in oocyte maturation during folliculogenesis. Front Cell Dev Biol. 2022;10:806890. 35433675 10.3389/fcell.2022.806890 PMC9009531

[16] Ruebel M, Shankar K, Gaddy D, Lindsey F, Badger T, Andres A. Maternal obesity is associated with ovarian inflammation and upregulation of early growth response factor 1. Am J Physiol Endocrinol Metab. 2016;311(1):E269-77. doi: 10.1152/ajpendo.00524.2015 27279249

[17] Wyse BA, Fuchs Weizman N, Defer M, Montbriand J, Szaraz P, Librach C. The follicular fluid adipocytokine milieu could serve as a prediction tool for fertility treatment outcomes. Reprod Biomed Online. 2021;43(4):738–46. doi: 10.1016/j.rbmo.2021.07.001 34474976

[18] Kim T, Kim Y, Lucien F, Zhao Y, Enninga EAL. Decreased gremlin 1 expression in women with BMI ≥35 kg/m2 is mediated by interleukin 10 and interleukin 1β in the follicular fluid. F S Sci. 2020;1(1):16–26. doi: 10.1016/j.xfss.2020.06.003 34296200 PMC8291741

[19] Dumesic DA, Meldrum DR, Katz-Jaffe MG, Krisher RL, Schoolcraft WB. Oocyte environment: follicular fluid and cumulus cells are critical for oocyte health. Fertil Steril. 2015;103(2):303–16. doi: 10.1016/j.fertnstert.2014.11.015 25497448

[20] Da Broi MG, Giorgi VSI, Wang F, Keefe DL, Albertini D, Navarro PA. Influence of follicular fluid and cumulus cells on oocyte quality: clinical implications. J Assist Reprod Genet. 2018;35(5):735–51. doi: 10.1007/s10815-018-1143-3 29497954 PMC5984887

[21] Viardot-Foucault V, Zhou J, Bi D, Takinami Y, Chan JKY, Lee YH. Dehydroepiandrosterone supplementation and the impact of follicular fluid metabolome and cytokinome profiles in poor ovarian responders. J Ovarian Res. 2023;16(1):107. doi: 10.1186/s13048-023-01166-6 37268990 PMC10239139

[22] Dunning KR, Russell DL, Robker RL. Lipids and oocyte developmental competence: the role of fatty acids and β-oxidation. Reproduction. 2014;148(1):R15–27. doi: 10.1530/REP-13-0251 24760880

[23] Perret BP, Parinaud J, Ribbes H, Moatti JP, Pontonnier G, Chap H, et al. Lipoprotein and phospholipid distribution in human follicular fluids. Fertil Steril. 1985;43(3):405–9. doi: 10.1016/s0015-0282(16)48440-2 3979578

[24] Gervais A, Battista M-C, Carranza-Mamane B, Lavoie HB, Baillargeon J-P. Follicular fluid concentrations of lipids and their metabolites are associated with intraovarian gonadotropin-stimulated androgen production in women undergoing in vitro fertilization. J Clin Endocrinol Metab. 2015;100(5):1845–54. doi: 10.1210/jc.2014-3649 25695883

[25] Batushansky A, Zacharia A, Shehadeh A, Bruck-Haimson R, Saidemberg D, Kogan NM, et al. A shift in glycerolipid metabolism defines the follicular fluid of IVF patients with unexplained infertility. Biomolecules. 2020;10(8). 10.3390/biom10081135 PMC7465802 32752038

[26] Zarezadeh R, Nouri M, Hamdi K, Shaaker M, Mehdizadeh A, Darabi M. Fatty acids of follicular fluid phospholipids and triglycerides display distinct association with IVF outcomes. Reprod Biomed Online. 2021;42(2):301–9. doi: 10.1016/j.rbmo.2020.09.024 33279420

[27] Liu Y, Tilleman K, Vlaeminck B, Gervais R, Chouinard PY, De Sutter P, et al. The fatty acid composition in follicles is related to the developmental potential of oocytes up to the blastocyst stage: a single-centre cohort study. Reprod Biol Endocrinol. 2022;20(1):107. doi: 10.1186/s12958-022-00974-7 35879714 PMC9310456

[28] Ruiz-Sanz J-I, Pérez-Ruiz I, Meijide S, Ferrando M, Larreategui Z, Ruiz-Larrea M-B. Lower follicular n-3 polyunsaturated fatty acid levels are associated with a better response to ovarian stimulation. J Assist Reprod Genet. 2019;36(3):473–82. doi: 10.1007/s10815-018-1384-1 30547270 PMC6439102

[29] Jungheim ES, Macones GA, Odem RR, Patterson BW, Lanzendorf SE, Ratts VS, et al. Associations between free fatty acids, cumulus oocyte complex morphology and ovarian function during in vitro fertilization. Fertil Steril. 2011;95(6):1970–4. doi: 10.1016/j.fertnstert.2011.01.154 21353671 PMC3080431

[30] Várnagy A, Bene J, Sulyok E, Kovács GL, Bódis J, Melegh B. Acylcarnitine esters profiling of serum and follicular fluid in patients undergoing in vitro fertilization. Reprod Biol Endocrinol. 2013;11:67. 23866102 10.1186/1477-7827-11-67 PMC3724743

[31] Yang J, Li Y, Li S, Zhang Y, Feng R, Huang R, et al. Metabolic signatures in human follicular fluid identify lysophosphatidylcholine as a predictor of follicular development. Commun Biol. 2022;5(1):763. doi: 10.1038/s42003-022-03710-4 35906399 PMC9334733

[32] Hood RB, Liang D, Tan Y, Ford JB, Souter I, Chavarro JE, et al. Serum and follicular fluid metabolome and markers of ovarian stimulation. Hum Reprod. 2023;38(11):2196–207. doi: 10.1093/humrep/dead189 37740688 PMC10628502

[33] Song J, Xiang S, Pang C, Guo J, Sun Z. Metabolomic alternations of follicular fluid of obese women undergoing in-vitro fertilization treatment. Sci Rep. 2020;10(1):5968. doi: 10.1038/s41598-020-62975-z 32249791 PMC7136245

[34] Valckx SD, De Pauw I, De Neubourg D, Inion I, Berth M, Fransen E, et al. BMI-related metabolic composition of the follicular fluid of women undergoing assisted reproductive treatment and the consequences for oocyte and embryo quality. Hum Reprod. 2012;27(12):3531–9. 23019302 10.1093/humrep/des350

[35] Valckx SDM, Arias-Alvarez M, De Pauw I, Fievez V, Vlaeminck B, Fransen E, et al. Fatty acid composition of the follicular fluid of normal weight, overweight and obese women undergoing assisted reproductive treatment: a descriptive cross-sectional study. Reprod Biol Endocrinol. 2014;12:13. doi: 10.1186/1477-7827-12-13 24498875 PMC3916060

[36] de Lima CB, Barbosa GZ, Ispada J, Dos Santos EC, Milazzotto MP. Lipid availability during in vitro maturation alters oocyte lipid content and blastocyst development and metabolism. Reprod Domest Anim. 2023;58(7):920–8. doi: 10.1111/rda.14367 37120750

[37] Pawlak P, Malyszka N, Szczerbal I, Kolodziejski P. Fatty acid induced lipolysis influences embryo development, gene expression and lipid droplet formation in the porcine cumulus cells†. Biol Reprod. 2020;103(1):36–48. doi: 10.1093/biolre/ioaa045 32318713 PMC7313259

[38] Ohata K, Ezoe K, Miki T, Kouraba S, Fujiwara N, Yabuuchi A, et al. Effects of fatty acid supplementation during vitrification and warming on the developmental competence of mouse, bovine and human oocytes and embryos. Reprod Biomed Online. 2021;43(1):14–25. doi: 10.1016/j.rbmo.2021.03.022 34049810

[39] Haam J-H, Kim BT, Kim EM, Kwon H, Kang J-H, Park JH, et al. Diagnosis of obesity: 2022 update of clinical practice guidelines for obesity by the Korean society for the study of obesity. J Obes Metab Syndr. 2023;32(2):121–9. doi: 10.7570/jomes23031 37386771 PMC10327686

[40] Matyash V, Liebisch G, Kurzchalia TV, Shevchenko A, Schwudke D. Lipid extraction by methyl-tert-butyl ether for high-throughput lipidomics. J Lipid Res. 2008;49(5):1137–46. doi: 10.1194/jlr.D700041-JLR200 18281723 PMC2311442

[41] Want EJ, Wilson ID, Gika H, Theodoridis G, Plumb RS, Shockcor J, et al. Global metabolic profiling procedures for urine using UPLC-MS. Nat Protoc. 2010;5(6):1005–18. 20448546 10.1038/nprot.2010.50

[42] Khan R, Jiang X, Hameed U, Shi Q. Role of lipid metabolism and signaling in mammalian oocyte maturation, quality, and acquisition of competence. Front Cell Dev Biol. 2021;9:639704. doi: 10.3389/fcell.2021.639704 33748128 PMC7973101

[43] Zhao J, Wang W, Zhang L, Zhang J, Sturmey R, Zhang J. Dynamic metabolism during early mammalian embryogenesis. Development. 2023;150(20):dev202148. doi: 10.1242/dev.202148 37877936

[44] Zhang L, Zhao J, Lam SM, Chen L, Gao Y, Wang W, et al. Low-input lipidomics reveals lipid metabolism remodelling during early mammalian embryo development. Nat Cell Biol. 2024;26(2):278–93. doi: 10.1038/s41556-023-01341-3 38302721

[45] Wallace M, Cottell E, Gibney MJ, McAuliffe FM, Wingfield M, Brennan L. An investigation into the relationship between the metabolic profile of follicular fluid, oocyte developmental potential, and implantation outcome. Fertil Steril. 2012;97(5):1078–84.e1-8. doi: 10.1016/j.fertnstert.2012.01.122 22365382

[46] Hofer P, Taschler U, Schreiber R, Kotzbeck P, Schoiswohl G. The lipolysome-a highly complex and dynamic protein network orchestrating cytoplasmic triacylglycerol degradation. Metabolites. 2020;10(4). 10.3390/metabo10040147 PMC7240967 32290093

[47] Duncan RE, Ahmadian M, Jaworski K, Sarkadi-Nagy E, Sul HS. Regulation of lipolysis in adipocytes. Annu Rev Nutr. 2007;27:79–101. doi: 10.1146/annurev.nutr.27.061406.093734 17313320 PMC2885771

[48] Anifandis G, Michopoulos A, Daponte A, Chatzimeletiou K, Simopoulou M, Messini CI, et al. Artificial oocyte activation: physiological, pathophysiological and ethical aspects. Syst Biol Reprod Med. 2019;65(1):3–11. doi: 10.1080/19396368.2018.1516000 30207496

[49] Kalive M, Faust JJ, Koeneman BA, Capco DG. Involvement of the PKC family in regulation of early development. Mol Reprod Dev. 2010;77(2):95–104. 19777543 10.1002/mrd.21112

[50] Yi Z-Y, Liang Q-X, Meng T-G, Li J, Dong M-Z, Hou Y, et al. PKCβ1 regulates meiotic cell cycle in mouse oocyte. Cell Cycle. 2019;18(4):395–412. doi: 10.1080/15384101.2018.1564492 30730241 PMC6422498

[51] Yu Y, Halet G, Lai FA, Swann K. Regulation of diacylglycerol production and protein kinase C stimulation during sperm- and PLCzeta-mediated mouse egg activation. Biol Cell. 2008;100(11):633–43. doi: 10.1042/BC20080033 18471090 PMC2615188

[52] Nair R, Manikkath J, Hegde AR, Mutalik S, Kalthur G, Adiga SK. Liposome-encapsulated diacyl glycerol and inositol triphosphate-induced delayed oocyte activation and poor development of parthenotes. J Turk Ger Gynecol Assoc. 2017;18(3):102–9. doi: 10.4274/jtgga.2017.0014 28890423 PMC5590204

[53] Lum MA, Barger CJ, Hsu AH, Leontieva OV, Black AR, Black JD. Protein Kinase Cα (PKCα) is resistant to long term desensitization/down-regulation by prolonged diacylglycerol stimulation. J Biol Chem. 2016;291(12):6331–46. doi: 10.1074/jbc.M115.696211 26769967 PMC4813581

[54] Kiley SC, Parker PJ, Fabbro D, Jaken S. Differential regulation of protein kinase C isozymes by thyrotropin-releasing hormone in GH4C1 cells. J Biol Chem. 1991;266(35):23761–8. doi: 10.1016/s0021-9258(18)54348-9 1748652

[55] Olivier AR, Parker PJ. Bombesin, platelet-derived growth factor, and diacylglycerol induce selective membrane association and down-regulation of protein kinase C isotypes in Swiss 3T3 cells. J Biol Chem. 1994;269(4):2758–63. doi: 10.1016/s0021-9258(17)42008-4 8300608

[56] Erion DM, Shulman GI. Diacylglycerol-mediated insulin resistance. Nat Med. 2010;16(4):400–2. doi: 10.1038/nm0410-400 20376053 PMC3730126

[57] Jani S, Da Eira D, Hadday I, Bikopoulos G, Mohasses A, de Pinho RA, et al. Distinct mechanisms involving diacylglycerol, ceramides, and inflammation underlie insulin resistance in oxidative and glycolytic muscles from high fat-fed rats. Sci Rep. 2021;11(1):19160. doi: 10.1038/s41598-021-98819-7 34580412 PMC8476522

[58] Petersen MC, Shulman GI. Roles of diacylglycerols and ceramides in hepatic insulin resistance. Trends Pharmacol Sci. 2017;38(7):649–65. doi: 10.1016/j.tips.2017.04.004 28551355 PMC5499157

[59] Pei Z, Deng K, Xu C, Zhang S. The molecular regulatory mechanisms of meiotic arrest and resumption in oocyte development and maturation. Reprod Biol Endocrinol. 2023;21(1):90. 37784186 10.1186/s12958-023-01143-0 PMC10544615

[60] Zheng D-M, An Z-N, Ge M-H, Wei D-Z, Jiang D-W, Xing X-J, et al. Medium & long-chain acylcarnitine’s relation to lipid metabolism as potential predictors for diabetic cardiomyopathy: a metabolomic study. Lipids Health Dis. 2021;20(1):151. doi: 10.1186/s12944-021-01576-9 34727932 PMC8562007

[61] Liang C, Zhang X, Qi C, Hu H, Zhang Q, Zhu X, et al. UHPLC-MS-MS analysis of oxylipins metabolomics components of follicular fluid in infertile individuals with diminished ovarian reserve. Reprod Biol Endocrinol. 2021;19(1):143. doi: 10.1186/s12958-021-00825-x 34521427 PMC8438979

[62] Liu Y, Tilleman K, Vlaeminck B, Gervais R, Chouinard PY, De Sutter P, et al. The fatty acid composition in follicles is related to the developmental potential of oocytes up to the blastocyst stage: a single-centre cohort study. Reprod Biol Endocrinol. 2022;20(1):107. doi: 10.1186/s12958-022-00974-7 35879714 PMC9310456

