# Supplementary material for: Impact of obesity on follicular fluid lipid composition and IVF/ICSI outcomes in Korean women: A lipidomic study
Source: PLoS One. 2025 May 23;20(5):e0324511. doi: 10.1371/journal.pone.0324511 (PMC12101671; doi:10.1371/journal.pone.0324511)
Supplement: S1 Table — SRM, selected reaction monitoring; LC, liquid chromatography; MS, mass spectrometry; LPC, lysophosphatidylcholine; LPE, lysophosphatidylethanolamine; PC, phosphatidylcholine; PE, phosphatidylethanolamine (DOCX) [file pone.0324511.s001.docx]

**S1 Table.** **Selected reaction monitoring (SRM) condition of phospholipids in lipid droplet by liquid chromatography-tandem mass spectrometry (LC-MS/MS)**

| No. | Compound | Adduct | Precursor ion (*m/z*) | Product ion (*m/z*) |  |
| --- | --- | --- | --- | --- | --- |
| 1 | LPC 16:1 | [M+H]^+^ | 494.3 | 184.1 |  |
| 2 | LPC 16:0 |  | 496.3 | 184.1 |  |
| 3 | LPC 18:3 |  | 518.3 | 184.1 |  |
| 4 | LPC 18:2 |  | 520.3 | 184.1 |  |
| 5 | LPC 18:1 |  | 522.3 | 184.1 |  |
| 6 | LPC 18:0 |  | 524.3 | 184.1 |  |
| 7 | LPC 20:5 |  | 542.3 | 184.1 |  |
| 8 | LPC 20:4 |  | 544.3 | 184.1 |  |
| 9 | LPC 20:3 |  | 546.3 | 184.1 |  |
| 10 | LPC 20:2 |  | 548.3 | 184.1 |  |
| 11 | LPC 20:1 |  | 550.3 | 184.1 |  |
| 12 | LPC 22:6 |  | 568.3 | 184.1 |  |
| 13 | LPC 22:5 |  | 570.3 | 184.1 |  |
| 14 | LPC 22:4 |  | 572.3 | 184.1 |  |
| 15 | LPE 16:0 | [M+H]^+^ | 454.3 | 313.2 |  |
| 16 | LPE 18:2 |  | 478.3 | 337.2 |  |
| 17 | LPE 18:1 |  | 480.3 | 339.2 |  |
| 18 | LPE 18:0 |  | 482.3 | 341.2 |  |
| 19 | LPE 20:5 |  | 500.3 | 359.2 |  |
| 20 | LPE 20:4 |  | 502.3 | 361.2 |  |
| 21 | LPE 20:3 |  | 504.3 | 363.2 |  |
| 22 | LPE 22:6 |  | 526.3 | 385.2 |  |
| 23 | LPE 22:5 |  | 528.3 | 387.2 |  |
| 24 | LPE 22:4 |  | 530.3 | 389.2 |  |
| 25 | PC 32:1 | [M+H]^+^ | 732.6 | 184.1 |  |
| 26 | PC 32:0 |  | 734.6 | 184.1 |  |
| 27 | PC 34:4 |  | 754.6 | 184.1 |  |
| 28 | PC 34:3 |  | 756.6 | 184.1 |  |
| 29 | PC 34:2 |  | 758.6 | 184.1 |  |
| 30 | PC 34:1 |  | 760.6 | 184.1 |  |
| 31 | PC 34:0 |  | 762.6 | 184.1 |  |
| 32 | PC 36:5 |  | 780.6 | 184.1 |  |
| 33 | PC 36:4 |  | 782.6 | 184.1 |  |
| 34 | PC 36:3 |  | 784.6 | 184.1 |  |
| 35 | | PC 36:2 |  | 786.6 | 184.1 |
| 36 | | PC 36:1 |  | 788.6 | 184.1 |
| 37 | | PC 36:0 |  | 790.6 | 184.1 |
| 38 | | PC 38:7 |  | 804.6 | 184.1 |
| 39 | | PC 38:6 |  | 806.6 | 184.1 |
| 40 | | PC 38:5 |  | 808.6 | 184.1 |
| 41 | | PC 38:4 |  | 810.6 | 184.1 |
| 42 | | PC 38:3 |  | 812.6 | 184.1 |
| 43 | | PC 38:2 |  | 814.6 | 184.1 |
| 44 | | PC 40:8 |  | 830.6 | 184.1 |
| 45 | | PC 40:7 |  | 832.6 | 184.1 |
| 46 | | PC 40:6 |  | 834.6 | 184.1 |
| 47 | | PC 40:5 |  | 836.6 | 184.1 |
| 48 | | PC 40:4 |  | 838.6 | 184.1 |
| 49 | | PE 34:0 | [M+H]^+^ | 720.6 | 579.5 |
| 50 | | PE 36:1 |  | 746.6 | 605.5 |
| 51 | PE 38:3 |  | 770.6 | 629.5 |  |
| 52 | PE 40:5 |  | 794.6 | 653.5 |  |
| 53 | PE 40:4 |  | 796.6 | 655.5 |  |
| 54 | Plasmanyl PC 40:8 | [M+H]^+^ | 816.6 | 184.1 |  |
| 55 | Plasmanyl PC 40:7 |  | 818.6 | 184.1 |  |
| 56 | Plasmenyl PC 32:1 | [M+H]^+^ | 716.6 | 184.1 |  |
| 57 | Plasmenyl PC 32:0 |  | 718.6 | 184.1 |  |
| 58 | Plasmenyl PC 34:3 |  | 740.6 | 184.1 |  |
| 59 | Plasmenyl PC 34:1 |  | 744.6 | 184.1 |  |
| 60 | Plasmenyl PC 34:0 |  | 746.6 | 184.1 |  |
| 61 | Plasmenyl PC 36:5 |  | 764.6 | 184.1 |  |
| 62 | Plasmenyl PC 36:4 |  | 766.6 | 184.1 |  |
| 63 | Plasmenyl PC 36:2 |  | 770.6 | 184.1 |  |
| 64 | Plasmenyl PC 38:6 |  | 790.6 | 184.1 |  |
| 65 | Plasmenyl PC 38:5 |  | 792.6 | 184.1 |  |
| 66 | Plasmenyl PC 38:4 |  | 794.6 | 184.1 |  |
| 67 | Plasmenyl PC 38:3 |  | 796.6 | 184.1 |  |
| 68 | Plasmenyl PC 40:7 |  | 816.6 | 184.1 |  |
| 69 | Plasmenyl PC 40:6 |  | 818.6 | 184.1 |  |
| 70 | Plasmenyl PC 40:5 |  | 820.6 | 184.1 |  |
| 71 | Plasmenyl PC 40:4 |  | 822.6 | 184.1 |  |
| 72 | Plasmenyl PE 34:2 (16:0/18:2) | [M+H]^+^ | 700.5 | 337.2 |  |
| 73 | Plasmenyl PE 34:1 (16:0/18:1) |  | 702.5 | 339.2 |  |
| 74 | Plasmenyl PE 34:0 (16:0/18:0) |  | 704.5 | 313.2 |  |
| 75 | Plasmenyl PE 36:5 (16:0/20:5) |  | 722.5 | 359.2 |  |
| 76 | Plasmenyl PE 36:4 (16:0/20:4) |  | 724.5 | 361.2 |  |
| 77 | Plasmenyl PE 36:3 (16:0/20:3) |  | 726.5 | 363.2 |  |
| 78 | Plasmenyl PE 36:2 (18:0/18:2) |  | 728.5 | 337.2 |  |
| 79 | Plasmenyl PE 36:1 (18:0/18:1) |  | 730.5 | 339.2 |  |
| 80 | Plasmenyl PE 38:6 (16:0/22:6) |  | 748.5 | 385.2 |  |
| 81 | Plasmenyl PE 38:5 (18:0/20:5) |  | 750.5 | 359.2 |  |
| 82 | Plasmenyl PE 38:5 (18:1/20:4) |  | 750.5 | 361.2 |  |
| 83 | Plasmenyl PE 38:4 (18:0/20:4) |  | 752.5 | 389.2 |  |
| 84 | Plasmenyl PE 38:4 (16:0/22:4) |  | 752.5 | 363.2 |  |
| 85 | Plasmenyl PE 38:3 (18:0/20:6) |  | 754.5 | 385.2 |  |
| 86 | Plasmenyl PE 40:7 (18:1/22:6) |  | 774.5 | 385.2 |  |
| 87 | Plasmenyl PE 40:6 (18:0/22:6) |  | 776.5 | 389.2 |  |

Lysophosphatidylcholine, LPC; Lysophosphatidylethanolamine, LPE; Phosphatidylcholine, PC; Phosphatidylethanolamine, PE
