# Supplementary material for: Impact of obesity on follicular fluid lipid composition and IVF/ICSI outcomes in Korean women: A lipidomic study
Source: PLoS One. 2025 May 23;20(5):e0324511. doi: 10.1371/journal.pone.0324511 (PMC12101671; doi:10.1371/journal.pone.0324511)
Supplement: S2 Table — SRM, selected reaction monitoring; LC, liquid chromatography; MS, mass spectrometry; MAG, monoacylglycerol; DAG, diacylglycerol; TAG, triacylglycerol. (DOCX) [file pone.0324511.s002.docx]

**S2 Table.** **Selected reaction monitoring (SRM) condition of neutral lipids in lipid droplet by liquid chromatography-tandem mass spectrometry (LC-MS/MS)**

| No. | Compound | Adduct | Precursor ion (*m/z*) | Product ion (*m/z*) |
| --- | --- | --- | --- | --- |
| 1 | MAG 16:0 | [M+H]^+^ | 348.3 | 313.3 |
| 2 | DAG 32:2 | [M+H]^+^ | 582.5 | 547.5 |
| 3 | DAG 32:1 |  | 584.5 | 549.5 |
| 4 | DAG 34:0 |  | 614.5 | 579.5 |
| 5 | DAG 36:5 |  | 632.5 | 597.5 |
| 6 | DAG 36:1 |  | 640.5 | 605.5 |
| 7 | TAG 46:2 (16:1/14:0/16:1) | [M+NH_4_]^+^ | 792.7 | 521.3 |
| 8 | TAG 46:1 (16:0/16:1/14:0) |  | 794.7 | 549.3 |
| 9 | TAG 46:0 (16:0/14:0/16:0) |  | 796.7 | 523.3 |
| 10 | TAG 48:3 (16:1/14:0/18:2) |  | 818.7 | 521.3 |
| 11 | TAG 48:2 (14:0/18:2/16:0) |  | 820.7 | 547.3 |
| 12 | TAG 48:1 (18:1/14:0/16:0) |  | 822.7 | 549.3 |
| 13 | TAG 48:0 (16:0/16:0/16:0) |  | 824.7 | 551.3 |
| 14 | TAG 50:4 (16:1/18:3/16:0) |  | 844.7 | 571.3 |
| 15 | TAG 50:3 (18:2/16:1/16:0) |  | 846.7 | 573.3 |
| 16 | TAG 50:2 (18:1/16:1/16:0) |  | 848.7 | 573.3 |
| 17 | TAG 50:1 (16:0/18:1/16:0) |  | 850.7 | 577.3 |
| 18 | TAG 50:0 (16:0/16:0/18:0) |  | 852.7 | 551.3 |
| 19 | TAG 52:6 (16:1/18:2/18:3) |  | 868.7 | 573.3 |
| 20 | TAG 52:5 (18:3/18:1/16:1) |  | 870.7 | 599.3 |
| 21 | TAG 52:4 (18:1/16:1/18:2) |  | 872.7 | 575.3 |
| 22 | TAG 52:3 (16:0/18:2/18:1) |  | 874.7 | 575.3 |
| 23 | TAG 52:2 (16:0/18:1/18:1) |  | 876.7 | 577.3 |
| 24 | TAG 52:1 (18:0/18:1/16:0) |  | 878.7 | 605.3 |
| 25 | TAG 54:7 (18:2/18:3/18:2) |  | 894.7 | 597.3 |
| 26 | TAG 54:6 (20:4/16:0/18:2) |  | 896.7 | 599.3 |
| 27 | TAG 54:5 (18:1/18:2/18:2) |  | 898.7 | 601.3 |
| 28 | TAG 54:4 (18:1/18:2/18:1) |  | 900.7 | 601.3 |
| 29 | TAG 54:3 (18:2/18:0/18:1) |  | 902.7 | 603.3 |
| 30 | TAG 54:2 (18:0/18:1/18:1) |  | 904.7 | 605.3 |
| 31 | TAG 54:1 (18:0/18:1/18:0) |  | 906.7 | 605.3 |
| 32 | TAG 56:8 (18:2/16:0/22:6) |  | 920.7 | 575.3 |
| 33 | TAG 56:7 (18:2/18:1/20:4) |  | 922.7 | 601.3 |
| 34 | TAG 56:6 (18:1/20:4/18:1) |  | 924.7 | 625.3 |
| 35 | TAG 56:5 (20:3/18:1/18:1) |  | 926.7 | 627.3 |
| 36 | TAG 56:4 (18:2/20:1/18:1) |  | 928.7 | 631.3 |
| 37 | TAG 56:3 (20:1/18:1/18:1) |  | 930.7 | 631.3 |
| 38 | TAG 56:2 (18:1/20:0/18:1) |  | 932.7 | 633.3 |
| 39 | TAG 58:9 (18:1/18:2/22:6) |  | 946.7 | 601.3 |
| 40 | TAG 58:8 (18:1/18:1/22:6) |  | 948.7 | 603.3 |
| 41 | TAG 58:7 (20:4/20:2/18:1) |  | 950.7 | 651.3 |
| 42 | TAG 58:6 (22:4/18:1/18:1) |  | 952.7 | 653.3 |
| 43 | TAG 58:5 (20:1/20:3/18:1) |  | 954.7 | 655.3 |

Monoacylglycerol, MAG; Diacylglycerol, DAG ; Triacylglycerol, TAG
