# Supplementary material for: Impact of obesity on follicular fluid lipid composition and IVF/ICSI outcomes in Korean women: A lipidomic study
Source: PLoS One. 2025 May 23;20(5):e0324511. doi: 10.1371/journal.pone.0324511 (PMC12101671; doi:10.1371/journal.pone.0324511)
Supplement: S3 Table — SRM, selected reaction monitoring; LC, liquid chromatography; MS, mass spectrometry; SM, sphingomyelin; CER, ceramide. (DOCX) [file pone.0324511.s003.docx]

**S3 Table.** **Selected reaction monitoring (SRM) condition of sphingolipid in lipid droplet by liquid chromatography-tandem mass spectrometry (LC-MS/MS)**

| No. | Compound | Adduct | Precursor ion (*m/z*) | Product ion (*m/z*) |
| --- | --- | --- | --- | --- |
| 1 | SM 32:1 | [M+H]^+^ | 675.6 | 184.1 |
| 2 | SM 32:0 |  | 577.6 | 184.1 |
| 3 | SM 34:1 |  | 703.6 | 184.1 |
| 4 | SM 34:0 |  | 705.6 | 184.1 |
| 5 | SM 36:3 |  | 727.6 | 184.1 |
| 6 | SM 36:2 |  | 729.6 | 184.1 |
| 7 | SM 36:1 |  | 731.6 | 184.1 |
| 8 | SM 36:0 |  | 733.6 | 184.1 |
| 9 | SM 38:2 |  | 757.6 | 184.1 |
| 10 | SM 40:8 |  | 773.6 | 184.1 |
| 11 | CER 38:5 (d18:1/20:4) | [M+H]^+^ | 586.5 | 264.3 |

Sphingomyelin, SM;Ceramide, CER
