# Supplementary material for: Impact of obesity on follicular fluid lipid composition and IVF/ICSI outcomes in Korean women: A lipidomic study
Source: PLoS One. 2025 May 23;20(5):e0324511. doi: 10.1371/journal.pone.0324511 (PMC12101671; doi:10.1371/journal.pone.0324511)
Supplement: S4 Table — SRM, selected reaction monitoring; LC, liquid chromatography; MS, mass spectrometry; CE, cholesteryl ester. (DOCX) [file pone.0324511.s004.docx]

**S4 Table.** **Selected reaction monitoring (SRM) condition of sterol lipid in lipid droplet by liquid chromatography-tandem mass spectrometry (LC-MS/MS)**

| No. | Compound | Adduct | Precursor ion (*m/z*) | Product ion (*m/z*) |
| --- | --- | --- | --- | --- |
| 1 | Cholesterol | [M+H]^+^ | 369.4 | 147.3 |
| 2 | CE 16:1 | [M+H]^+^ | 640.6 | 369.3 |
| 3 | CE 16:0 |  | 642.6 | 369.3 |
| 4 | CE 18:3 |  | 664.6 | 369.3 |
| 5 | CE 18:2 |  | 666.6 | 369.3 |
| 6 | CE 18:0 |  | 670.6 | 369.3 |
| 7 | CE 20:5 |  | 688.6 | 369.3 |
| 8 | CE 20:4 |  | 690.6 | 369.3 |
| 9 | CE 22:6 |  | 714.6 | 369.3 |
| 10 | CE 22:5 |  | 716.6 | 369.3 |

Cholesteryl ester, CE;
