# Supplementary material for: Impact of obesity on follicular fluid lipid composition and IVF/ICSI outcomes in Korean women: A lipidomic study
Source: PLoS One. 2025 May 23;20(5):e0324511. doi: 10.1371/journal.pone.0324511 (PMC12101671; doi:10.1371/journal.pone.0324511)
Supplement: S5 Table — SRM, selected reaction monitoring; LC, liquid chromatography; MS, mass spectrometry. (DOCX) [file pone.0324511.s005.docx]

**S5 Table. Selected reaction monitoring (SRM) condition of carnitine in lipid droplet by liquid chromatography-tandem mass spectrometry (LC-MS/MS)**

| No. | Compound | Adduct | Precursor ion (*m/z*) | Product ion (*m/z*) |
| --- | --- | --- | --- | --- |
| 1 | Acylcarnitine 2:0 | [M+H]^+^ | 204.3 | 85.1 |
| 2 | Acylcarnitine 3:0 |  | 218.3 | 85.1 |
| 3 | Acylcarnitine 4:0 |  | 232.3 | 85.1 |
| 4 | Acylcarnitine 16:1 |  | 398.3 | 85.1 |
| 5 | Acylcarnitine 16:0 |  | 400.3 | 85.1 |
| 6 | Acylcarnitine 18:2 |  | 424.4 | 85.1 |
| 7 | Acylcarnitine 18:1 |  | 426.4 | 85.1 |
| 8 | Acylcarnitine 18:0 |  | 428.4 | 85.1 |
